# Predictors of wasting among children under-five years in largely food insecure area of north Wollo, Ethiopia: a cross-sectional study

**DOI:** 10.1017/jns.2022.8

**Published:** 2022-02-09

**Authors:** Anchamo Anato

**Affiliations:** Department of Human Nutrition, School of Nutrition, Food Science and Technology, Hawassa University, Hawassa, Ethiopia

**Keywords:** Associated factors, Northern Ethiopia, Prevalence, Under-five children, Wasting

## Abstract

Child undernutrition is widespread in low- and middle-income countries and is linked with weakened immunity and increased risks of morbidity and mortality. Ethiopia has made a marked reduction in stunting, but there has, however, been little progress in wasting reduction and limited evidence in food insecure areas may hamper the design of effective interventions. Therefore, the aim of the present study was to determine the contributing factors to persistent high prevalence of wasting among 6–59-month-old children. A community-based cross-sectional study was employed in February to March 2020, and included 384 mother–child pairs. Data were collected using a structured interviewer-administered questionnaire. Bivariate and multivariable logistic regression analyses were conducted. The overall prevalence of wasting was 12⋅8 % (95 % CI 9⋅1, 16⋅1); with 5⋅8 % severely wasted. Factors significantly associated with wasting were child age 6–23 (*v.* 24–59 months), delayed initiation of breast-feeding, diarrhoeal illness in the last 2 weeks, poor dietary diversity and low socioeconomic status. The present findings support that aligning poverty reduction interventions and healthcare services is important to accelerate wasting reduction more equitably and achieve the World Health Assembly's target and SDG goal #2 in the coming years. Improving accessibility and affordability of nutritious foods and early diagnosis and treatment of childhood morbidity are critical to address childhood wasting in the context of food insecure areas.

## Background

Globally, in 2020, 45⋅4 million children younger than 5 years of age were wasted; of these, 13⋅6 million were severely wasted^(1)^. The highest prevalence of wasting is found in Asia (69 %) followed by Africa (27 %). In low- and middle-income countries (LMICs), wasting is estimated to contribute to about 800 000 deaths per year in children younger than 5 years of age, with about 60 % attributable to severe wasting^(2)^. Undernutrition is an underlying cause of nearly half (45 %) of the deaths in children under 5 years of age worldwide^(3)^ and is associated with reduced immunity and impaired cognitive function of children^(4)^ leading to loss of future productivity, overall poor health outcomes and poor school performance^(5)^.

Despite the world's remarkable progress of undernutrition reduction in children under 5 years of age, wasting in many Sub-Saharan African countries remains high. Ethiopia has made substantial progress in stunting reduction, from 58 % in 2000 to 37 % in 2019^(6)^. There has, however, been little progress in wasting reduction, from 12 % in 2000 to 7⋅2 % in 2019, indicating that Ethiopia is not on-course to achieve the World Health Assembly (WHA) targets of reducing and maintaining wasting prevalence to <5 % by 2025^(7)^. The prevalence of wasting reduction is not uniform across different regions of Ethiopia and varies from 16⋅8 % in Amhara to 21⋅1 % in Somali^(6,8)^. The causes of wasting can be complex and include child morbidities and poor feeding practices^(9)^. Several studies have investigated determinants of wasting^(10,11)^. Findings showed that child sex, age^(12)^, diversified diet, number of less than five children^(13)^, delayed initiation of breast-feeding, low socieconomic status^(14,15)^ and child morbidity^(16)^ were associated with wasting.

Recognising the extent and grave consequences of child undernutrition, the Ethiopia government has been implementing multiple nutrition intervention programmes. Nevertheless, the prevalence of wasting is still high in Ethiopia, and there has been insufficient progress in wasting reduction. Unfortunately, the impact of stunting reduction interventions will be difficult to realise without improvements in the proportion of wasted children as stunting and wasting often occur in the same population and share many similar causal factors^(17)^. Growing evidence that stunting and wasting are linked and that wasting can increase the risk of subsequent stunting^(18)^ has important programmatic and policy implications. However, the potential of context-based factors, if considered and integrated in multiple nutrition intervention programmes, to result in substantial wasting reduction and support to meet the targets remains largely unknown. Therefore, the aim of the present study was to determine the contributing factors to the high prevalence of wasting in food insecure rural area in Amhara, Ethiopia. The findings of the study may provide policy makers and implementers priorities for focus to make rapid reductions in wasting and to improve the on-going nutrition interventions in Ethiopia.

## Methods and materials

### Study design, setting, participants and period

**Fig. 1.**
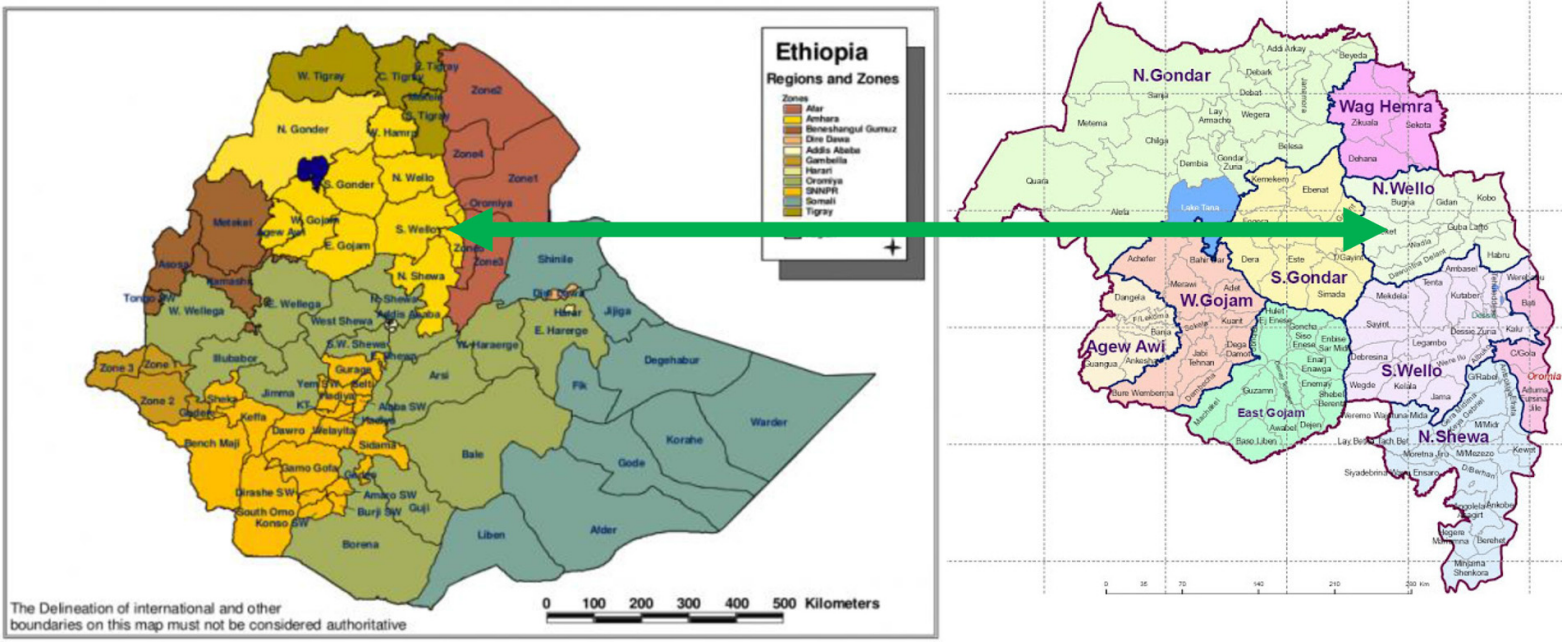
Regional map of Ethiopia showing the study site, in north Wollo, Ethiopia.

A community-based cross-sectional study was conducted to assess determinants of wasting among children aged 6–59 months of age in three food insecure districts (Sekota, Lasta and Meket) in north Ethiopia from February to March 2020 (Fig. 1). From the three districts, fifteen *kebeles* (the smallest administrative unit in Ethiopia) were selected randomly. In the region, the prevalence of undernutrition was high with stunting (41⋅3 %), underweight (26⋅7 %) and wasting (7⋅6 %) that exceeded that of the national average^(6)^. The inhabitants of the region mainly produce maize (*Zea mays* L.), millet (*Pennisetum glaucum*), pulses and teff (*Eragrostis tef*) as staple foods. Vegetables such as kale and potato are also grown. Traditional animal rearing such as cattle is common, mainly as a source of income.

### Sample size, population and sampling procedures

The sample size of 392 was calculated using the single population proportion formula with assumptions of 18 % wasting in the Amhara region^(12)^, a 95 % confidence interval, 5 % margin of error, ~15 % non-response rate and a design effect of 1⋅5. After excluding incomplete data from the total sample, 384 participants were included in the final analysis. Each *kebele* (the lowest administrative unit in Ethiopia) was considered as a cluster. All mothers with children aged 6–59 months and who had lived at least 6 months in the selected *kebeles* were completed from the database that was compiled by the research team prior to actual data collection. The number of mother–child pairs to be selected was proportionally allocated to the fifteen *kebeles* in the three districts based on the total number of the households with 6–59-month-old children in each *kebele*. The study participants were then selected by systematic random sampling from this sampling frame. For households with two or more children in the desired age range, one was selected to participate using the lottery method. The mothers/caregivers of these children were interviewed by the trained data collectors.

### Outcome variable

The outcome variable for the present study was wasting among children 6–59 months of age. Wasting (acute undernutrition) was defined as a weight-for-length/height *z*-score <–2sd and severe wasting as a weight-for-length/height *z*-score <–3sd^(19)^.

### Exclusion criteria

Children with physical disabilities and severe illnesses were excluded from the present study.

### Data collection instrument and quality control

A structured and pretested questionnaire was used to assess determinants of wasting. Basic socio-demographic characteristics such as maternal and child age, occupation, maternal educational status, marital status, place of delivery, antenatal care services, family size, number of under-five children at the household, breast-feeding patterns, use of family planning, farm land size, estimated average household monthly income, ownership of sanitary facility, source of drinking water, child age in months, child sex and morbidity were collected. Child morbidity was reported by the mother if the child had morbidity during the 2 weeks prior to the data collection. The questionnaire was prepared using simple and easily understandable Amharic language and administered using the local language via trained and experienced data collectors fluent in both languages. The instrument was pretested in the locality and appropriate customisation and modifications had been made. The reliability and content validity of the tool have also been evaluated. Accordingly, items with reliability coefficients less than 0⋅7 kappa were amended or removed.

Data on dietary intake were collected in the home using a single 24-h recall with the mother of each child. The method used in the present study is adapted and validated for use in developing countries^(20)^. Each mother was asked to recall all foods and fluids consumed by a child in the previous 24 h including time, type of meal, ingredients used, amount of total dish and amount consumed. After completing the interview, the foods consumed were categorised into seven food groups such as (1) grains, roots and tubers; (2) legumes and nuts; (3) dairy products; (4) eggs; (5) flesh foods (meat, fish, poultry and organ meats); (6) vitamin-rich fruits and vegetables; and (7) other fruits and vegetables. The proportion of children consuming ≥4 food groups was considered as good diversified diet and <4 as poor^(21)^. Early initiation of breast-feeding, prelacteal feeding and vaccination status of the child were also assessed.

Household wealth index was constructed from nineteen variables related to ownership of selected assets using principal component analysis (PCA). The components with Eigen values greater than one were retained to construct the wealth index and the households were then categorised as low, medium or upper based on their composite scores.

Child length/height was measured to the nearest 0⋅1 cm using a portable length/stature measuring board (Perspective Enterprises, Portage, MI, USA). Weight was measured with an electronic balance (UNICEF Seca 770). Measurements of recumbent length (for <24 months of age) or standing height (for ≥24 months) and weight of children were taken in duplicate using calibrated equipment and standardised techniques using standardised procedures^(22)^, to the nearest 0⋅1 cm or 0⋅01 kg, respectively, with children wearing light clothes and barefoot. A third measurement was taken if the difference between the first two measurements was outside the allowable difference (weight = 100 g, and length/height = 5 mm).

### Data processing and analysis

Data completeness was checked and then coded and entered into SPSS v. 22. Anthropometric data was calculated using WHO anthro and classified as wasted if weight-for-length/weight-for-height *z*-score <–2sd^(21)^. Frequency, percentage, mean and standard deviation were computed as descriptive statistics. Multicollinearity was checked by a variance inflation factor (VIF) and non-collinear variables were entered one by one into a bivariate logistic regression model to identify candidate variable for the multivariate logistic regression model. Those variables with *P* < 0⋅25 in bivariate regression were included in multivariate logistic regression^(23)^. Finally, adjusted odds ratio (AORs) with *P* < 0⋅05 and 95 % CI were calculated and reported.

### Ethical consideration

Ethical clearance was obtained from Hawassa University Institutional Review Board (Ref. No. IRB/178/10). The purpose of the study was explained in a formal letter to district administration and then written approval was obtained from district health offices. Prior to enrolment in the study, the purpose of the study was explained and informed written consent was obtained from mothers/caregivers.

## Results

### Socio-demographic characteristics of study participants

Socio-demographic characteristics of the mothers–child pairs are presented in Table 1. Of the 392 mothers–child pairs recruited for the study, 384 mothers agreed and participated in the study (97⋅4 % response rate). The mean age (±sd) of the mothers was 28⋅3±(5⋅5) years. Most of the mothers in the present study were married (90⋅4 %); 68 % had no formal education. About one-third (32⋅8 %) of mothers were housewives, and the average number of persons per household was 4⋅85. The mean age (±sd) of the children was 22⋅7 ± 13⋅1 months. Among the children, 59⋅4 % were males and 40⋅6 % were females.

**Table 1.** Socio-demographic and economic characteristics of study participants from Amhara region, 2020 (*n* = 384)

Variables	*n*	%	Mean (sd)
Father has some formal education	130	33⋅9	
Maternal characteristics			
Age, years			28⋅3 (5⋅5)
No education	261	68⋅0	
Some formal education	123	32⋅0	
Housewife	126	32⋅8	
Farmer	186	48⋅4	
Others^a^	72	18⋅7	
Married	347	90⋅4	
Separated	31	8⋅1	
Widowed	6	1⋅6	
Child characteristics			
Age, months			22⋅7 (13⋅1)
Proportion male	228	59⋅4	
Household characteristics			
Family size			4⋅9 (1⋅6)
Number of under-five children			1⋅5 (0⋅6)
Estimated monthly income (Eth. Birr)			906⋅2 (2075⋅1)
Owns livestock	270	70⋅3	
Owns chickens	27	7⋅0	
Protected drinking water source			
Protected	213	55⋅5	
Wealth index^b^			
Poor	176	45⋅8	
Middle	149	38⋅8	
Upper	59	15⋅4	

a Daily labourer/government employee/petty trade.

b Wealth index is constructed using principal component analysis (PCA).

### Child caring practices, child morbidity and maternal health services seeking behaviours

Two hundred and sixty-four children (68⋅8 %) had been fed breast milk within 1 h after birth.

More than one-third (35⋅2 %) of children had symptoms of acute respiratory infection and about 38⋅1 % had diarrhoeal morbidity during the previous 2 weeks prior to survey. Only 88 % of children had received at least one vaccination for his/her age. Most children (80⋅5 %) had poor dietary diversity (<4 food groups from seven groups). More than half (57⋅9 %) of the mothers had never used any family planning. More than half (58⋅5 %) of mother reported home delivery; 59⋅9 % had antenatal care at least once during index child pregnancy. About 47⋅1 % households owned sanitary facility such as toilet.

### Prevalence of wasting

**Fig. 2.**
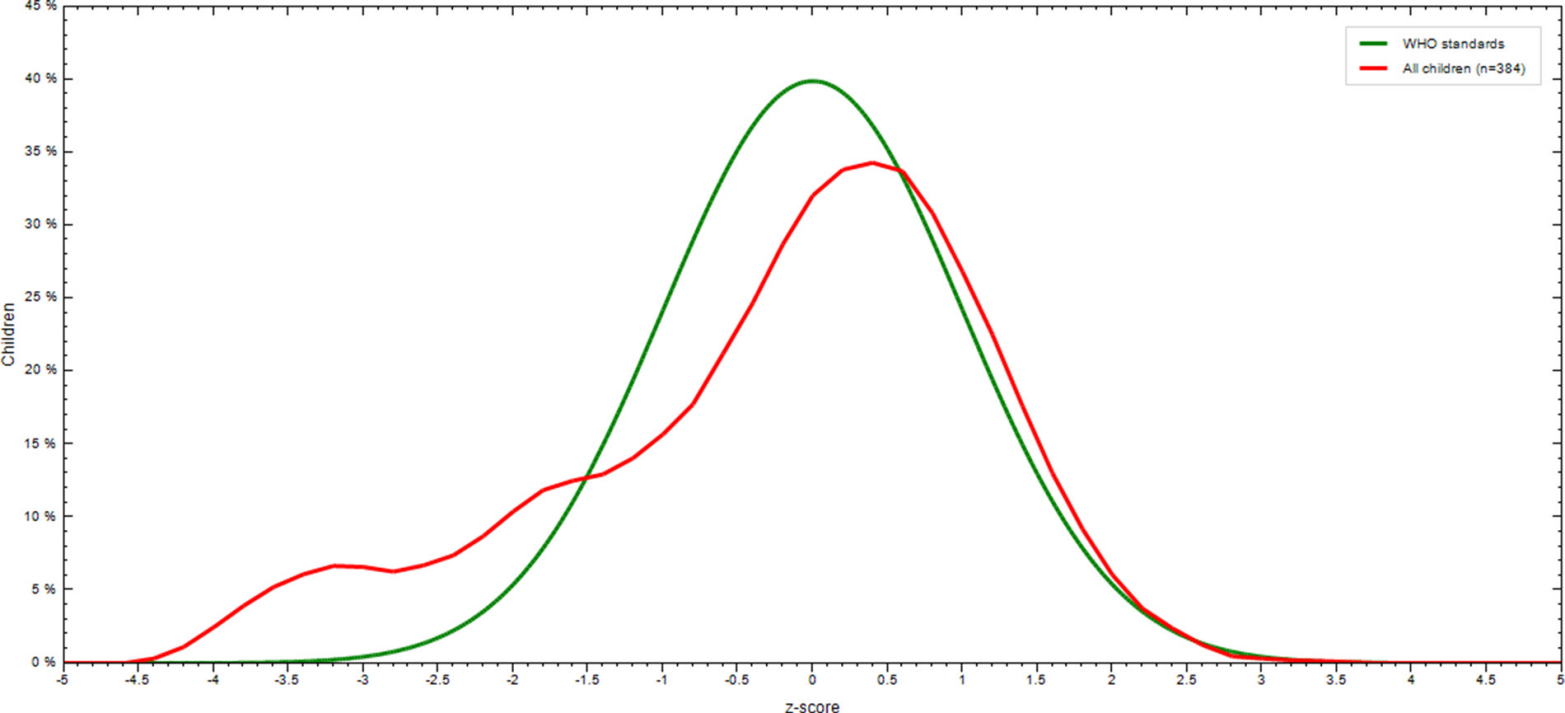
WL/HZ score of children 6–59 months of age compared with the WHO growth standards in north Wollo, Ethiopia.

In the present study, the prevalence of wasting was 12⋅8 % (95 % CI 9⋅1, 16⋅1), of this number, 7⋅0 and 5⋅8 % were moderately and severely wasted, respectively. Mean ± sd of weight-for-height *z*-score was −0⋅27 ± 1⋅42. As shown in Fig. 2, *z*-score curves are displaced to the left of the WHO child growth standard curve depicting that wasting is prevalent among children in the study area.

### Determinants of wasting

Table 2 presents the results of adjusted logistic regressions for factors associated with wasting. In adjusted logistic regression models, delayed initiation of breast-feeding after delivery (AOR 2⋅11, 95 % CI 1⋅05, 4⋅23), diarrhoeal illness in the 2 weeks prior to the survey (AOR 2⋅02, 95 % CI 1⋅03, 3⋅96), poor diversified diet (AOR 2⋅93, 95 % CI 1⋅09, 8⋅82) and low socioeconomic status (AOR 2⋅32, 95 % CI 1⋅16, 4⋅62) were significantly associated with wasting. Relative to 24–59-month-old children, increased odds of wasting were found among children 6–23 months of age (AOR 3⋅39, 95 % CI 1⋅63, 7⋅07).

**Table 2.** Factors associated with wasting among under-five children (*n* = 384) in Amhara region, Ethiopia

Variables	Number of wasted children		COR (95 % CI)	AOR (95 % CI)
	No	Yes		
Child age (months)				
6–23	165	38	3⋅55 (1⋅85, 8⋅22)	3⋅39 (1⋅63, 7⋅07)*
24–59	170	11	1	1
Sex of child				
Female	139	17	1	1
Male	196	32	1⋅36 (0⋅84, 2⋅21)	0⋅73 (0⋅37, 1⋅44)
Paternal education				
Some education	119	10	1	1
Not educated	216	39	2⋅15 (1⋅04, 4⋅46)	1⋅86 (0⋅76, 4⋅55)
Maternal education				
Some education	113	10	1	1
Not educated	222	39	1⋅98 (0⋅98, 4⋅12)	0⋅66 (0⋅28, 1⋅56)
Initiation of BF				
Immediately	239	20	1	1
After 1 h	96	29	2⋅39 (1⋅30, 4⋅39)	2⋅11 (1⋅05, 4⋅23)*
Diarrhoeal morbidity in the last 2 weeks				
Yes	119	27	2⋅12 (1⋅15, 3⋅89)	2⋅02 (1⋅03, 3⋅96)*
No	216	21	1	1
Received diversified diet				
Poor (<4 food groups)	264	45	3⋅03 (1⋅05, 8⋅69)	2⋅93 (1⋅09, 8⋅82)*
Good (≥4 food groups)	71	4	1	
Attended ANC				
Yes	206	24	1	1
No	129	25	1⋅66 (0⋅91, 3⋅04)	1⋅11 (0⋅55, 2⋅22)
Wealth index				
Poor	143	33	2⋅76 (1⋅47, 5⋅23)	2⋅32 (1⋅16, 4⋅62)*
Medium/rich	192	16	1	1
Source of drinking water				
Improved	190	23	1	
Not improved	145	26	1⋅48 (0⋅81, 2⋅71)	1⋅17 (0⋅57, 2⋅39)
Household waste disposal				
Proper	158	22	1	
Open field	177	27	1⋅09 (0⋅60, 2⋅01)	0⋅89 (0⋅44, 1⋅79)

AOR, adjusted odd ratio; COR, crude odd ratio.

* *P* < 0.05.

## Discussion

Despite efforts of many entities in Ethiopia, the rates of wasting remain unacceptably high; in the present study, the prevalence of wasting was 12⋅8 % (95 % CI 9⋅1, 16⋅1). From the multivariable logistic regression model, child's age, delayed initiation of breast-feeding, diarrhoeal infection in the 2 weeks prior to the survey, poor dietary diversity and low socioeconomic status were significantly associated with wasting.

In the present study, the age of child had a significant effect on prevalence of wasting. The odds of wasting were 3⋅39 times higher among children age 6–23 months as compared to children age 24–59 months. Similar results were reported previously in Ethiopia and Senegal^(15,24)^. This may happen because younger children (6–23 months) have a smaller gastric capacity compared with the older children (24–59 months), and they are in a transition phase from breast milk dominated feeding to complementary feeding. The first 2 years of life is a critical period characterised by high requirements of energy and micronutrients for rapid physical growth and mental development^(25)^. However, in rural areas of Ethiopia, the actual complementary foods that young children consume are predominately unrefined cereal-based diets that are low in energy and nutrients^(26,27)^. This suggests that ensuring availability, accessibility and affordability of nutrient-dense foods for all is needed to significantly tackle wasting during the complementary feeding period.

Delayed initiation of breast-feeding was found to be significantly associated with increased odds of wasting, which is in parallel with studies reported in Ethiopia and elsewhere^(28,29)^. Breast-feeding during the first hour after birth is critical to improve health and nutritional status of children as well as to reduce the risk of childhood morbidities^(30)^. Early breast-feeding is one of the ideal nutrition interventions which can save many lives in low-income countries^(28)^. Although national infant and young feeding (IYCF) guidelines recommend that every newborn baby should start breast-feeding immediately after birth, in the present study, nearly one-third (31⋅3 %) of the children were not breast-feed as per recommendation. Recent studies from Ethiopia have shown that colostrum feeding practices are too low and many mothers discard colostrum, partly due to traditional beliefs towards the fluid^(31,32)^. Lack of timely counselling from health extension workers and poor maternal knowledge and misperceptions^(33)^ also can be related to sub-optimal breast-feeding practices. Thus, the provision of nutrition education on health and nutritional benefits of optimal child feeding practices through health extension packages could improve child feeding practices and accelerate undernutrition reduction.

In the present study, diarrhoeal morbidity in the 2 weeks prior to the survey had a significant effect on acute undernutrition of children. The odds of the wasting were 2-fold higher among children who had diarrhoeal morbidity compared with those who had no such morbidity in the last 2 weeks before the survey. These results were congruent with the findings of studies conducted in other regions of Ethiopia^(34,35)^ and in Nigeria and Pakistan^(36,37)^. Diarrhoeal infection is associated with increased loss of body fluids, nutrients and electrolytes along with poor appetite and impaired nutrient absorption leading to increased risk of acute undernutrition. This finding suggests that in addition to undernutrition reduction interventions, investments in diarrhoea prevention, control and early treatment would support and complement accelerated undernutrition reduction programmes in Ethiopia. Aligning nutrition interventions and health systems is thus needed, if the WHA targets of achieving wasting <5 % by 2025 and the government's plan (Seqota Declaration) to end stunting by 2030, as well as achieving SDG#2 by 2030 are to be realised.

Besides health status of children, a key determinant of wasting was the quality of children's diets^(12,14)^. Consistent with other reports, the present study revealed that the odds of wasting were 2⋅9 times higher among children who did not receive a diversified diet compared with those who had better quality diets (≥4 food groups). In the present study, only 19⋅5 % of children consumed four or more food groups in the previous 24 h prior to the survey. This is unfortunate, as consumption of diversified diet is a proxy indicator of diet quality and nutrient adequacy of a child's diet^(38,39)^ and is consistently associated with improved nutritional status of infants and young children^(40)^. A diversified diet is essential to fulfill high requirements of energy and micronutrients to sustain rapid physical growth and development during early childhood and is an effective intervention to tackle undernutrition^(41)^. Optimal complementary feeding is critical to reduce undernutrition^(42)^, but low coverage of complementary feeding interventions in developing countries, poor maternal literacy about optimal breast-feeding and complementary feeding practices^(43,44)^, as well as limited production of diverse foods and lack of accessibility and affordability of nutrient-dense foods can be constraints associated with sub-optimal complementary feeding^(45,46)^. Although sup-optimal complementary feeding practice is common in Sub-Saharan Africa, children from the poorest households are the most affected^(47)^.

The odds of wasting were three times higher among children from lower socioeconomic household than those of upper wealth status. The higher prevalence of wasting among lower socioeconomic status is not unexpected and was consistent with earlier literature on the determinants of wasting among children <5 years of age^(48,49)^. In Ethiopia, as in many developing countries, low-income households may suffer from high prices, seasonal fluctuation or unavailability of nutrient-dense foods that can limit the households to reliance on predominantly cereal-based and unhealthy diets, with little or no animal source foods, vegetables and fruits^(50,51)^. For example, Heady and Alderman^(52)^ showed that the prices of nutrient-dense foods, particularly, fruits and animal source foods have been increased much more than the other food groups in developing countries, including Ethiopia. Indeed, in many low-income countries, the poorest segments of the population not only suffer from high food prices and associated poor feeding practices, but also from inequalities in education and health services. Poorly paid jobs and temporary employment can trap them in a vicious cycle of poverty, poor health and high rates of child undernutrition^(53)^.

The present study has a number of limitations that need to be considered when interpreting the findings. First, data on child morbidity and feeding practices data relied on maternal recall which could have social desirability and recall bias. Second, the cross-sectional nature of the study does not allow causal inference relationship to be made; thus, the relations between child feeding practices and diarrhoeal morbidity, socioeconomic status and wasting should be considered as associations. Third, data collection period was a very short and thus finding may be affected by seasonal variations. Future detailed studies within and between years can help to understand the seasonal patterns in the prevalence of wasting and causal factors to inform programmes and policy to design effective interventions to prevent wasting.

Despite the above limitations, the present study is community-based with adequate sample size which achieved a 97⋅4 % response rate. The findings came from three districts and thus can be extrapolated to all districts of north Wollo, Ethiopia. Moreover, the prevalence of wasting and the five factors identified in the present study were in close agreement with that of the national study^(54)^_,_ suggesting that the findings from the present study can be generalised to the entire Ethiopia population.

## Conclusion

Acute undernutrition expressed as wasting is a public health problem in the study area. The prevalence of wasting was higher than the national average. Children's age, delayed initiation of breast-feeding, diarrhoeal infection in the 2 weeks prior to data collection, poor diversification of diets and low socioeconomic status were independent determinants of wasting among children <5 years age. The prevalence of wasting was uneven between wealth quintiles; the poorest population segments were disproportionately affected. The national progress in wasting reduction in children <5 years of age has not been enough and more work needs to be done in the upcoming years if Ethiopia is to achieve the WHA 2025 targets and Seqota Declaration^(55)^. Thus, aligning and strengthening poverty reduction interventions and primary healthcare services is needed to accelerate reduction in child undernutrition. Particular attention to increasing access to alternative sources of nutritious foods, to optimal complementary feeding practices, and to early diagnosis and treatment of childhood morbidity in the context of food insecure areas is critical. Furthermore, improving households’ income may be important to improve child feeding practices and to prevent acute undernutrition.
